# Dynamic Hydrogels in Breast Tumor Models

**DOI:** 10.3390/gels11110855

**Published:** 2025-10-26

**Authors:** Girdhari Rijal, In-Woo Park

**Affiliations:** 1 Department of Medical Laboratory Sciences, Public Health and Nutrition Sciences, School of Health and Clinical Professions, Division of Health Sciences, Fort Worth Campus, Tarleton State University, Crowley, TX 76036, USA; 2 Department of Microbiology, Immunology & Genetics, College of Biomedical and Translation Sciences, University of North Texas, Fort Worth, TX 76107, USA; inwoo.park@unthsc.edu

**Keywords:** dynamic hydrogel, breast cancer model, scaffold, drug delivery, biocompatibility, biodegradability, stimulus-responsive hydrogel

## Abstract

Fabricating breast tumor models that mimic the natural breast tissue-like microenvironment (normal or cancerous) both physically and bio-metabolically, despite extended research, is still a challenge. A native-mimicking breast tumor model is the demand since complex biophysiological mechanisms in the native breast tissue hinder deciphering the root causes of cancer initiation and progression. Hydrogels, which mimic the natural extracellular matrix (ECM), are increasingly demanded for various biomedical applications, including tissue engineering and tumor modeling. Their biomimetic 3D network structures have demonstrated significant potential to enhance the breast tumor model, treatment, and recovery. Additionally, 3D tumor organoids cultivated within hydrogels maintain the physical and genetic traits of native tumors, offering valuable platforms for personalized medicine and therapy response evaluation. Hydrogels are broadly classified into static and dynamic hydrogels. Static hydrogels, however, are inert to external stimuli and do not actively participate in biological processes or provide scaffolding systems. Dynamic hydrogels, on the other hand, adapt and respond to the surrounding microenvironment or even create new microenvironments according to physiological cues. Dynamic hydrogels typically involve reversible molecular interactions—through covalent or non-covalent bonds—enabling the fabrication of hydrogels tailored to meet the mechanical and physiological properties of target tissues. Although both static and dynamic hydrogels can be advanced by incorporating active nanomaterials, their combinations with dynamic hydrogels provide enhanced functionalities compared to static hydrogels. Further, engineered hydrogels with adipogenic and angiogenic properties support tissue integration and regeneration. Hydrogels also serve as efficient delivery systems for chemotherapeutic and immunotherapeutic agents, enabling localized, sustained release at tumor sites. This approach enhances therapeutic efficacy while minimizing systemic side effects, supporting ongoing research into hydrogel-based breast cancer therapies and reconstructive solutions. This review summarizes the roles of dynamic hydrogels in breast tumor models. Furthermore, this paper discusses the advantages of integrating nanoparticles with dynamic hydrogels for drug delivery, cancer treatment, and other biomedical applications, alongside the challenges and future perspectives.

## 1. Introduction

Hydrogels, polymeric materials characterized by high-water content and tissue-like elasticity, are emerging as promising tools in breast tumor models and their application in breast cancer therapy [1]. They offer novel possibilities for post-mastectomy reconstruction, patient-specific tumor modeling, and targeted drug delivery, enhancing treatment precision and reducing systemic toxicity. Hydrogels are rapidly advancing clinically, with over 100 hydrogel-based products approved by the FDA and EMA, primarily in tissue regeneration, highlighting their safety and versatility. Silicone-based implants remain FDA-approved standards for breast reconstruction, but hydrogels such as chitosan, alginate, and PEG are common in practice in a variety of breast tumor models through many modifications. For example, adipose-derived stem cells in GelMA scaffolds highlight hydrogel applicability for breast tissue regeneration [2,3,4].

**Figure 1:**
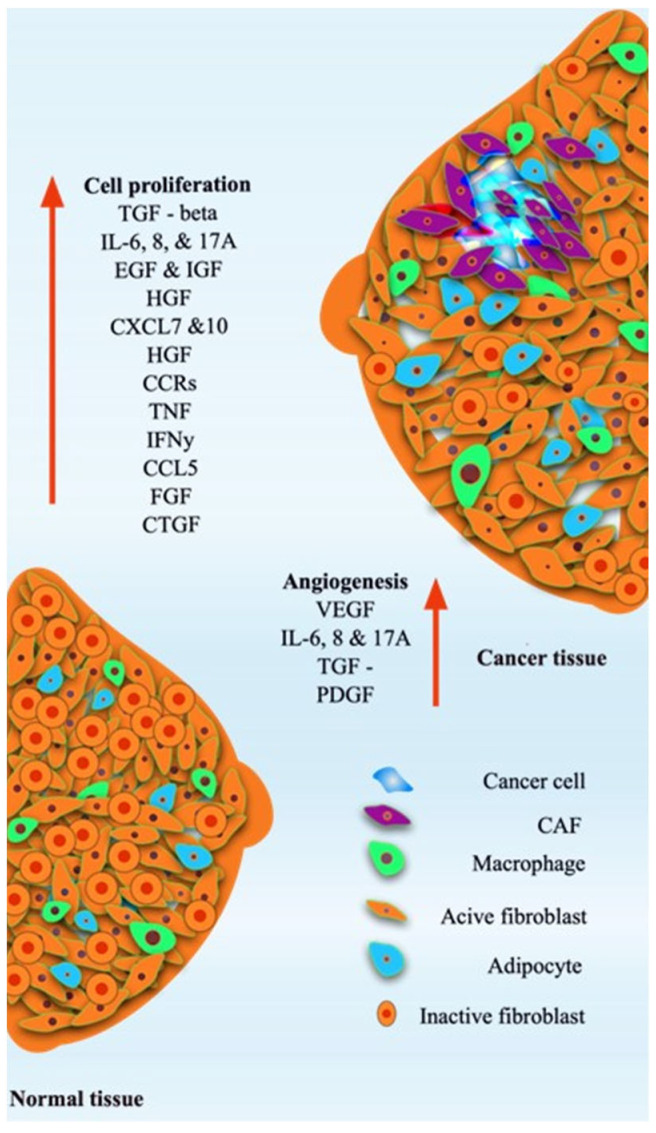
Breast tissue cells: normal vs. cancer. Cell proliferation and angiogenesis process are controlled in the normal tissue, whereas they are uncontrolled in the cancerous tissue because of the high expression of the angiogenesis and cell proliferation factors, as shown in the figure. Red arrows indicate the increase in the expression of cell proliferation and angiogenesis factors in cancer tissue compared to normal tissue.

Breast tissue has a complex structure with mammary glands that challenge the fabrication of the appropriate breast model. The mammary gland is composed of multiple layers of endothelial tissue, abundant adipose tissue encased within a basement membrane, and contains essential components such as blood vessels, lymphatic vessels, and stromal cells [5] (Figure 1). Angiogenesis factors such as VEGF, IL-6, IL-8, 17A, and TGF-PDGF and cell proliferation factors such as TGF -β, IL-6, IL-8, 17A, EGF, IGF, HGF, CXCL7 and 10, HGF, CCRs, INF, IFN-y, CCL5, FGF, and CTGF are responsible for the maintenance of the normal tissue. However, those factors are uncontrolled, leading to breast tissue growth with the direction of the cancer cells and CAFs (cancer-associated fibroblasts) [5,6] (Figure 1).

Breast cancer typically initiates with ductal hyperplasia and gradually progresses to a malignant tumor under persistent exposure to carcinogenic stimuli. The tumor microenvironment (TME), particularly the extracellular matrix (ECM) and macrophages, plays a pivotal role in supporting tumor initiation, progression, and invasion. Cellular heterogeneity observed in breast tissues is linked to the developmental origin of normal breast tissue and is exacerbated by changes in genetic and phenotypic traits during continuous proliferation and differentiation [6,7]. While normal somatic cells are regulated by stringent growth control mechanisms, mutations in tumor suppressor genes and the activation of telomerase allow cancer cells to evade these controls and replicate indefinitely [8]. Additionally, estrogen exposure is known to cause DNA damage and mutagenesis, further increasing breast cancer risk, particularly when immune surveillance mechanisms fail due to genetic mutations or signaling disruptions [9,10]. Metastasis, responsible for over 90% of cancer-related deaths, allows cancer cells to disseminate from the primary tumor through lymphatic and vascular routes, eventually colonizing organs such as bone, brain, liver, and lungs, with bone being the most frequent site in metastatic breast cancer [11]. Understanding this phenomenon, using hydrogels might be possible in the future after extensive advancements in dynamic hydrogels.

## 2. Breast Tumor Models

Tumor progression involves dynamic interactions between cancer cells and the tumor microenvironment (TME), including stromal fibroblasts, immune infiltrates, extracellular matrix (ECM) components, vascular networks, and soluble signaling molecules (Figure 1). Understanding these multifaceted interactions requires experimental models capable of accurately recapitulating both cellular and microenvironmental complexity.

The development and refinement of breast tumor models have been central to preclinical research, enabling studies of tumor initiation, progression, metastasis, and therapeutic response (Table 1). These models serve as indispensable tools for identifying molecular drivers, screening novel therapeutics, studying tumor–stroma and tumor–immune interactions, and understanding mechanisms of therapy resistance. However, no single model fully recapitulates all aspects of human breast cancer biology. Each system offers specific advantages and limitations, influencing its suitability for particular experimental goals. Therefore, careful classification and critical evaluation of available models are necessary to guide researchers in selecting appropriate platforms for their studies [12] (Table 1).

**Figure 2:**
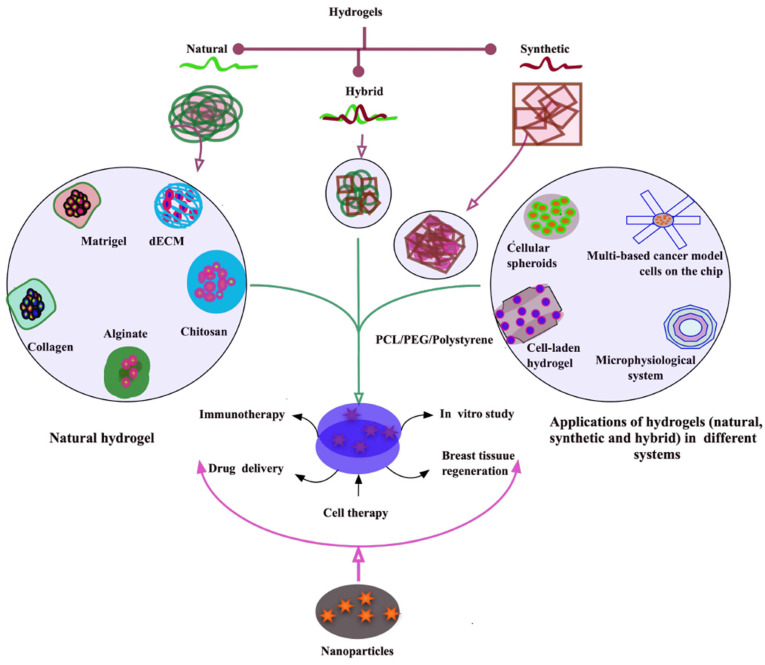
Hydrogels and their types and application in breast tumor models. Natural hydrogels (Matrigel, dECM, collagen, alginate, and chitosan), synthetic hydrogels from PCL, PECG, and polystyrene), and hybrid hydrogels, a combination of different biomaterials has been implicated in breast tumor models with/without nanoparticles along with different application model systems for various purposes, for example, cell therapy. Hydrogels are applied through various models in breast tumor models (e.g., cellular spheroids or tumoroids, cell-laden 3D hydrogel, micro-physiological systema, and chip methods).

Experimental models for breast cancer can be broadly classified into in vitro, ex vivo, and in vivo systems, with emerging approaches integrating bioengineering and computational modeling [13]. In vitro models include traditional two-dimensional (2D) cell culture systems, three-dimensional (3D) spheroids and organoids, co-culture systems, and microfluidic “tumor-on-a-chip” platforms [14]. These systems provide controlled, reproducible environments for mechanistic and high-throughput studies but vary significantly in their ability to replicate tumor architecture, ECM interactions, nutrient and oxygen gradients, and cellular heterogeneity. Two-dimensional cultures are particularly valuable for genetic manipulation, biochemical assays, and drug screening due to their simplicity and reproducibility [15]. However, they fail to reproduce critical aspects of tumor biology, including cell polarity, 3D cell–cell and cell–ECM interactions, and dynamic gradients of metabolites and oxygen [15]. In contrast, 3D cultures and organoid systems better mimic the native tissue architecture and support the emergence of clinically relevant phenotypes, such as cancer stem cell enrichment and invasion patterns [16]. Hydrogel-based scaffolds, including natural (e.g., Matrigel, collagen, hyaluronic acid) and synthetic (e.g., PEG-based) materials, provide tunable mechanical and biochemical cues to guide tumor growth and cell behavior [17] (Figure 2). Despite these advantages, 3D systems are technically more demanding, may suffer from batch-to-batch variability, and generally lack full vascularization and immune components.

Ex vivo approaches, such as precision-cut tumor slices and patient-derived explants, maintain native tissue architecture, ECM composition, and multicellular heterogeneity [18]. These models allow direct testing of therapeutic agents in a physiologically relevant context and enable investigation of cell–cell and cell–matrix interactions. However, ex vivo systems are limited by short viability, typically a few days, and low throughput. Additionally, logistical constraints related to the availability and processing of fresh human tumor tissue limit their broad applicability [18].

**Table 1:** Hydrogels in breast tumor models.

Hydrogel System	Material/Composition	Key Features	Applications in Breast Tumor Models	Limitations	References
Matrigel	Basement membrane extract (laminin, collagen IV, growth factors)	Mimics ECM, promotes cell adhesion, supports 3D organoid formation	Breast cancer spheroids/organoids, invasion studies, drug screening	Batch variability, animal-derived, poorly defined composition, limited mechanical tunability	[19,20,21]
Collagen-based hydrogels	Type I/III collagen	Biocompatible, fibrillar network, tunable stiffness	3D breast tumor culture, migration/invasion assays, angiogenesis studies	Limited long-term stability, low reproducibility due to source variation	[20,22]
Alginate	Polysaccharide from algae	Tunable stiffness, ionic crosslinking, low immunogenicity	3D breast spheroids, encapsulation of tumor and stromal cells, drug delivery studies	Poor cell adhesion unless modified, limited bioactive signals	[21,23,24]
Gelatin/GelMA (gelatin methacryloyl)	Denatured collagen, photopolymerizable	Cell-adhesive, tunable stiffness, light-crosslinkable	3D breast tumor spheroids, co-culture with fibroblasts/endothelial cells, drug response studies	Requires UV or photo-initiator, mechanical properties may differ from native ECM	[25,26]
Hyaluronic acid (HA) hydrogels	HA, often crosslinked	Mimics tumor ECM, supports proliferation/migration, interacts with CD44	Breast cancer invasion, CSC enrichment, drug testing	Mechanical properties may be limited; crosslinking can alter bioactivity	[27,28]
PEG-based hydrogels (polyethylene glycol)	Synthetic polymer, often functionalized	Chemically defined, tunable stiffness, degradable linkers	3D breast cancer culture, controlled drug delivery, mechanotransduction studies	Bio-inert without modification; requires functionalization for cell adhesion	[29,30]
Fibrin hydrogels	Fibrinogen + thrombin	Supports angiogenesis, tumor–stroma interactions	Breast tumor spheroids, vascularized tumor models, metastasis assays	Rapid degradation, batch variability, limited long-term culture	[20,31]
Chitosan-based hydrogels	Chitosan polysaccharide, sometimes blended with collagen or gelatin	Biodegradable, modifiable, supports 3D culture	3D breast tumor culture, drug screening, scaffold for co-culture	Poor mechanical strength alone; variable cell adhesion without modification	[20,32,33]
Synthetic hybrid hydrogels	Combinations: PEG + gelatin, HA + PEG, alginate + ECM proteins	Combines tunable mechanics with bioactivity	Personalized tumor organoids, mechanobiology studies, drug testing	More complex to fabricate; may require multi-step crosslinking	[34,35]

In vivo models remain essential for studying systemic tumor biology, including metastasis, tumor–immune interactions, and therapeutic responses. Xenograft models, including cell line-derived xenografts (CDXs) and patient-derived xenografts (PDXs), are widely employed to evaluate drug efficacy and mimic human tumor biology. PDX models preserve histopathological and genetic features of the donor tumor, enabling the study of patient-specific responses [36]. However, the requirement for immunodeficient hosts limits the ability to study immune interactions, and selective engraftment of aggressive tumors may introduce bias. Syngeneic models, which involve transplantation of mouse tumor cells into immunocompetent mice, allow investigation of tumor–immune dynamics and immunotherapies but are limited by interspecies differences that constrain translation to human disease [37]. Genetically engineered mouse models (GEMMs) provide spontaneous tumor formation under defined genetic alterations, recapitulating tumor initiation, progression, and metastasis in an intact microenvironment. While GEMMs offer valuable insights into tumor biology, they are expensive, time-consuming, and often represent specific genetic alterations rather than the heterogeneity observed in human tumors. Humanized mouse models, which integrate patient-derived tumors with reconstituted human immune systems, provide a promising platform for immuno-oncology studies [38]. Despite their translational potential, these models remain technically challenging, costly, and prone to incomplete immune reconstitution.

Emerging bioengineered platforms are further expanding the repertoire of breast tumor models. Three-dimensional bioprinting enables precise spatial control over tumor architecture, incorporation of stromal and vascular components, and the creation of customizable microenvironments [39]. Hydrogel-based dynamic scaffolds can mimic matrix stiffness, degradability, and biochemical gradients, providing more physiologically relevant conditions. Microfluidic platforms allow the integration of flow, vascular channels, and compartmentalized co-cultures, facilitating studies of invasion, intravasation, extravasation, and drug delivery [40]. Computational models complement experimental systems by simulating tumor growth dynamics, drug penetration, and pharmacokinetics, although they require high-quality input data and experimental validation [41].

A critical evaluation of these models reveals that no single system fully captures the complexity of human breast tumors. In vitro systems provide high-throughput, mechanistic insights but lack systemic context. Ex vivo models retain tissue architecture but are limited by duration and throughput. In vivo models capture systemic interactions but face species-specific translational barriers. Bioengineered and computational approaches offer innovative solutions but are constrained by technical complexity and validation requirements. The selection of an appropriate model depends on the specific research question, balancing biological relevance, reproducibility, and feasibility. Integrative approaches combining multiple complementary systems are increasingly adopted to enhance translational fidelity and predictive accuracy. As technological advances continue to refine tumor models, integrating multi-dimensional approaches that capture cellular heterogeneity, ECM dynamics, immune interactions, and systemic physiology will be critical for advancing breast cancer research and therapeutic development.

## 3. General Characteristics of Hydrogels

### 3.1. Natural Hydrogels

Derived from animal, plant, algal, or human tissues, natural hydrogels exhibit superior biocompatibility and support breast tissue regeneration [42]. A few natural hydrogels, common for breast tumor models, are herewith briefly discussed. Matrigel, dECM, collagen, alginate, and chitosan are used more commonly in breast tumor models with or without a composite with nanoparticles for various purposes, for example, immunotherapy, drug delivery, cell therapy, and breast tissue regeneration. Unique structures of biomaterials provide the special microenvironmental–scaffolding–physiological function to breast cancer cells (Figure 2).

Collagen, the predominant ECM protein in human breast tissue, along with decellularized extracellular matrix (dECM) and gelatin, plays a central role in promoting acinar structure formation, adipocyte proliferation, and epithelial branching [43,44]. dECM, composed of fibrillar proteins like collagens and elastin, and glycoproteins such as laminin and fibronectin, has been recognized as a biologically relevant scaffold that supports cell signaling, adhesion, and growth factor interactions [45]. The retention of the vascular network and ECM structure after decellularization provides a natural microenvironment conducive to cancer cell growth, making dECM an attractive material for in vitro tumor modeling [46]. The structural and biochemical integrity of dECM is highly dependent on the source tissue and the decellularization method, necessitating precise protocol selection based on tissue characteristics such as cell content, lipid density, and thickness. dECM derived from various animal and human tumor tissues has enabled the development of diverse tumor models, including porcine breast tissue-based 3D metastatic breast cancer and fibroblast cultures enriched with microfilament fragments [47,48]. Additionally, porcine kidney-derived dECM scaffolds, enhanced via chemical crosslinking, have been utilized to culture MCF-7 breast cancer cells and improve scaffold mechanics for tumor model applications [49,50] (Figure 2).

Gelatin, a hydrolysate of collagen composed mainly of glycine, proline, and hydroxyproline, exhibits excellent immunogenicity, hydrophilicity, and temperature sensitivity, making it a widely used material in tissue-engineered scaffolds (Figure 2). When dissolved in water, gelatin forms a viscous solution that transitions into a hydrogel upon cooling, enabling high-resolution scaffold fabrication through 3D printing techniques [51,52]. Its ability to reduce the viscosity and shear stress of bio-inks enhances printability and minimizes cellular damage during cell-laden bioprinting, supporting the development of effective tumor models. For instance, gelatin-based scaffolds have been shown to support breast cancer cell attachment and proliferation, with tunable porosity and mechanical properties that closely mimic the extracellular matrix of breast tumors. Moreover, gelatin can be chemically modified by crosslinking with polyethylene glycol and functional peptides, such as YIGSR or VEGF-mimetic sequences, to regulate cell binding and growth factor signaling, offering a versatile platform for cancer research and therapeutic testing. Methacrylamide-modified gelatin (GelMA) is favored for its tunable mechanics and physiological crosslinking ability [53,54]. Innovations combining GelMA with bioactive components such as calcium silicate (CaSiO_3_) or methacrylated κappa-carrageenan (CarMA) via 3D printing have yielded scaffolds that support adipogenesis, angiogenesis, and native tissue mimicry [55]. Nonetheless, traditional GelMA formulations generate excess free radicals and heterogeneous polymer networks, prompting alternatives like GelNB/Gel-SH photo-click scaffolds offering improved homogeneity and reduced cytotoxicity [56]. Integration of alginate-based microbeads and 3D printing further enabled the creation of porous, vascularized constructs resembling fatty human tissue [57]. Challenges with natural hydrogels include scalability, mechanical strength limitations, cost, and immune responses, which may be mitigated by hybridizing natural with synthetic polymers.

### 3.2. Synthetic Hydrogels

Synthetic hydrogels composed of polymers such as polyethylene glycol (PEG), polylactic-co-glycolic acid (PLGA), polycaprolactone (PCL), polyacrylamide (PAM), polyvinyl alcohol (PVA), and perfluorocarbons (PFs) offer tunable properties, enhanced mechanical strength, and durability ideal for breast tissue engineering [51] (Figure 2). PCL-based hydrogels have been extensively studied for their ability to replicate native tissue mechanics and support volumetric regeneration using 3D printing methods like fused filament fabrication and selective laser sintering. Composite scaffolds combining PCL with biomaterials such as collagen or platelet-rich plasma (PRP) demonstrated improved adipogenesis, vascularization, and integration in animal models [58]. Poly(2-hydroxyethyl methacrylate) (PHEMA)-based hydrogels show promise as breast implant materials due to their mechanical performance, self-recovery, and compressive properties similar to silicone prostheses [59]. Multi-component composites such as PVA/collagen/polypropylene glycol (PPG) exhibit enhanced mechanical strength, thermal stability, and cell compatibility via chemical crosslinking [60]. Despite these advantages, synthetic hydrogels face challenges in biocompatibility and fabrication complexity [61]. Further research on degradation kinetics, immune interactions, and clinical translation is essential.

Sodium alginate (SA) is a natural polymer derived from brown algae and composed of β-D-mannuronic acid and α-L-guluronic acid, and is widely used in tissue engineering due to its biocompatibility and ability to form hydrogels through ionic crosslinking with divalent cations [62]. In tumor model construction, SA serves as a substitute for glycosaminoglycans and is effective for immobilizing proteins and cells, making it a valuable scaffold material. Crosslinking agents such as calcium chloride (CaCl_2_) are commonly used to control the mechanical strength and structural integrity of SA-based hydrogels, although variations in SA concentration do not significantly influence cell growth or proliferation [63]. Studies using gelatin/SA composite hydrogels fabricated via 3D printing have shown that increasing the SA concentration improves scaffold elasticity and reduces surface roughness without affecting biological performance [64]. These findings highlight SA’s excellent mechanical tunability and biocompatibility, reinforcing its suitability for in vitro tumor modeling applications.

### 3.3. Composite or Hybrid Hydrogels

Hybrid hydrogels represent a versatile class of biomaterials with multiple definitions, encompassing (i) complexes formed via chemical or physical crosslinking of nanogels, (ii) polymer-based systems integrated with functional nanoparticles such as plasmonic, magnetic, or carbonaceous materials, and (iii) composites of chemically, functionally, and morphologically distinct building blocks derived from at least two molecular classes, including polysaccharides, proteins, peptides, or nano-/microstructures, synthetic biomaterials linked through physical or chemical interactions. Hybridization can occur at molecular or microscopic scales, determined by the size and nature of the constituents [1,42] (Figure 2).

These systems have been pioneered to address the inherent limitations of both natural and synthetic polymers, including the mechanical fragility and immunogenicity of natural materials and the limited biocompatibility and biodegradability of synthetic ones (Figure 2). By combining components from both sources, hybrid hydrogels achieve robust mechanical performance with favorable biocompatibility and degradability. For example, hyaluronic acid (HA)-based injectable hydrogels often suffer from rapid enzymatic degradation and mechanical weakness, limiting their use in load-bearing tissue engineering, while pure collagen hydrogels exhibit inadequate physical strength (Figure 2). Conversely, synthetic hydrogels such as poly (ethylene glycol) (PEG) lack intrinsic bioactivity, which can be introduced through the incorporation of bioactive natural materials [1,65].

Advances in modification strategies, including Schiff base and Diels–Alder reactions, diverse crosslinking techniques, and the integration of nano-/microstructures, have enabled precise tuning of mechanical, chemical, and biological properties (Figure 2). Emerging fabrication methods such as click chemistry, 3D printing, and photopatterning further expand their potential. Controlled release mechanisms for bioactive molecules enhance their therapeutic efficacy, positioning hybrid hydrogels as promising candidates for applications in therapeutic delivery, regenerative medicine, and next-generation biomedical technologies [1,23].

### 3.4. Static vs. Dynamic Hydrogels

Most synthetic hydrogels are classified as static due to their irreversible covalent crosslinks—mediated by Schiff base, Michael addition, or enzymatic interactions—that prevent responsiveness to environmental changes [66]. On the other hand, dynamic hydrogels are characterized by reversible covalent or supramolecular bonds that allow adaptation to environmental stimuli such as pH, temperature, light, mechanical forces, or specific enzymes [66,67] (Figure 2). These stimuli-responsive properties confer self-healing, shape-memory, tunable surface energy, and adjustable mechanical attributes such as viscoelasticity and porosity. Molecules in dynamic hydrogels typically engage in reversible disulfide, imine, boronate ester bonds, or non-covalent hydrophobic/hydrogen bonds, enabling injectability and self-healing suitable for clinical applications [68,69] (Table 2). Irreversible dynamic hydrogels degrade enzymatically or via pH changes, facilitating cell proliferation and migration. Both physical and chemical crosslinked hydrogels can form interpenetrating polymer networks (IPNs), enhancing mechanical strength and cellular functionality [66]. The incorporation of matrix fillers further extends mechanical robustness and imparts shear-thinning properties conducive to injectability [70]. Nanoparticles incorporated into dynamic hydrogels enhance sustained drug and cell release in vivo [71,72] (Figure 2).

## 4. Biomaterial Characteristics of Hydrogels

### 4.1. Biodegradability and Bioadhesion

**Figure 3:**
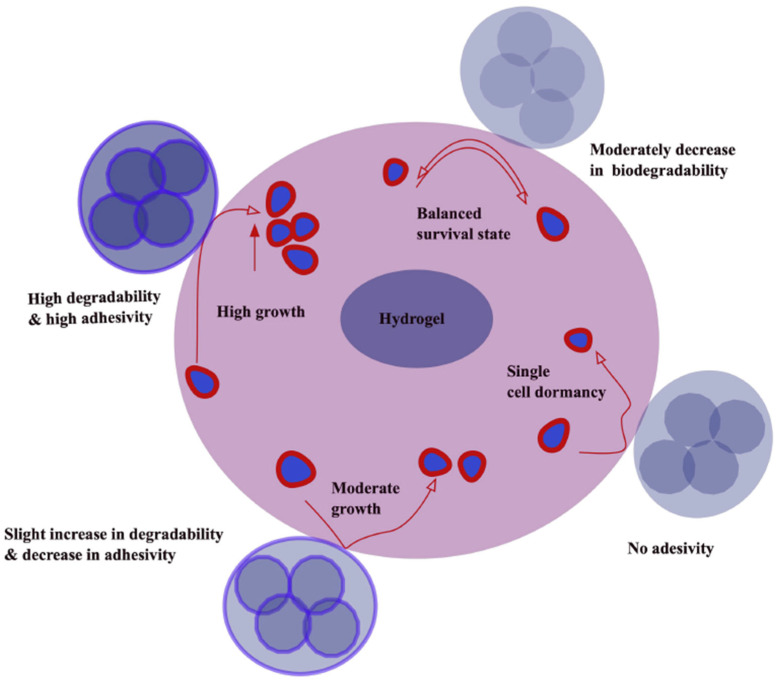
Biodegradability and adhesivity of the hydrogels and their scaffolds. Biodegradability and adhesivity of the hydrogel/scaffold determine not only the scaffolding properties, but also the effective release of cells and drugs, proliferation and growth, single cell dormancy, cell survival state.

Injectability is vital for dynamic hydrogels, allowing minimally invasive delivery. Hydrogel degradability influences cell metabolism and sprouting. And increasing matrix degradation enhances proangiogenic factor secretion while shaping distinct secretory profiles (Figure 3).

Matrix degradation is essential for wound healing, while excessive MMP activity contributes to chronic nonhealing wounds. In an ex vivo burn wound model, EC-MSC spheroid-loaded degradable and non-degradable gels both restored the stratified epidermal architecture. Non-degradable (PEG-DT) gels showed greater epidermal detachment than partially degradable (GPQ-A:PEG-DT) gels, although wound diameter was similar across treatments [73].

**Table 2:** Static vs. dynamic hydrogels.

S.N.	Feature	Static Hydrogels	Dynamic Hydrogels	References
1	Mechanical properties	Stable, stiff, with limited adaptability	Tunable, can remodel or self-heal in response to stimuli	[74,75,76]
2	Crosslinking and examples	Permanent, Covalent: PEGGA/PEG-NB, polyacrylamide (2D), GElMA at fixed DoF Physical: Collagen I (neutralized), Matrigel (batch variable), alginate-Ca^2+^	Reversible, dynamic covalent: hydrazone/oxime, boronate–diol, disulfide, thiol–ene with secondary light steps Supramolecular: B-cyclodextrin–adamantane, host–guest peptides, MMP-degradable PEG, photodegradable o-nitrobenzyl linkers, or weak supramolecular interactions	[77,78,79,80]
3	Mechanobiology	Good for static stiffness response curves (YAP/TAZ, focal adhesions). Limited stress relaxation control unless tailored	Stress relaxation and creep tunable, real-time stiffening (fibrosis) or softening (matrix degradation); supports durotaxis and mechanoadaptation studies	[81,82]
4	Biomimicry	Less biomimetic, static structure	Closer to natural ECM, adaptable and dynamic	[76,83]
5				
6	Stimuli responsiveness	Generally non-responsive	Response to pH, temperature, enzymes, light, redox, etc.	[76,84]
7	Self-healing ability	Absent (PEGDA/PAAm) or minimal (unless collagen/Matrigel)	Present, due to reversible crosslinking MMP-cleavable peptides (GPQGIWGQ, etc.)	[85,86,87]
8	Degradation	Controlled mainly by hydrolysis or enzymatic breakdown	Can degrade or restructure dynamically based on stimuli	[75,88]
9	Applications	Long-term implants, scaffolds needing stability	Drug delivery, tissue engineering, wound healing, 4D bioprinting	[89,90]
10	Advantages	High stability, mechanical robustness, simple, inexpensive, clear controls, batch-to-batch tunable (except Matrigel)	High adaptability, dynamic interactions, self-healing, physiologically closer to breast TME, captures progression, dormancy > reactivation, metastasis-like programs	[91,92]
11	Limitations	Lack of adaptability, no self-healing	Lower mechanical strength, sometimes unstable long term	[76,93]
12	Co-culture and TME complexity	Possible, but matrix lacks adaptive feedback to cells	Supports cell-driven desmoplasia, immune infiltration dynamics	[94,95]
13	Spatial/temporal patterning	Mostly pre-set, patterning requires multi-step fabrication	In situ photopatterning of stiffness/ligands; sequential cue delivery (e.g., EGF gradient after stiffening)	[96,97]
14	Drug testing	Stable baselines for screening doxorubicin, paclitaxel, tamoxifen, etc., good reproducibility	Can model acquire resistance by inducing progressive stiffening, HA accrual, or hypoxia formation, better for combination therapy timing studies	[98,99]

Cell adhesion regulates proliferation, migration, and differentiation. Optimal adhesion varies by application; tissue scaffolds require high adhesion, whereas blood contact devices need minimal adhesion. Incorporation of ECM-derived ligands such as RGD and RGD-modified commercial hydrogels demonstrate strong cell adhesion [100,101]. Strong bioadhesion, especially mucoadhesion, ensures prolonged tissue contact. Adhesion arises from polymer swelling, mucin interpenetration, and chemical bonding, influenced by polymer crosslink density, chain mobility, and functional groups (hydroxyl, carboxyl, thiol, catechol) [102,103,104]. Catechol-functionalized hydrogels, inspired by mussel adhesive proteins, exhibit strong underwater adhesion through quinone-mediated crosslinks. Examples include polydopamine-enriched hydrogels with guar gum showing robust skin adhesion. Dopamine-modified hyaluronic acid and polydopamine-coated graphene oxide enhance mucoadhesion and wound healing [105,106]. ECM-mimetic hydrogels using dopamine-modified gelatin and methacrylated chondroitin sulfate adhere tightly via boronate ester and thioether bonds [66]. Catechol-modified ε-poly-L-lysine hydrogels crosslinked with Fe^3+^ and oxidized dextran demonstrate repeatable adhesion with excellent cytocompatibility [107,108]. Underwater adhesive hydrogels and PEG–catechol systems containing ureidopyrimidinone motifs provide wound sealing with minimal inflammation.

Composite chitosan hydrogels with whitlockite nanoparticles improve coagulation via ion release, reducing bleeding more effectively than commercial products [109,110]. Boronate ester and imine bond crosslinking also enhance adhesion strength, suitable for surgical sealants resisting physiological pressures [111]. Reversible and painless detachment is achievable using temperature, pH, or chemical triggers, allowing controlled removal ideal for temporary biomedical applications [112,113]. Janus hydrogels with asymmetric adhesive properties provide tissue adhesion on one side and antifouling on the other, promising for tissue repair and other therapies [114].

### 4.2. Cell Aggregation Prevention

**Figure 4:**
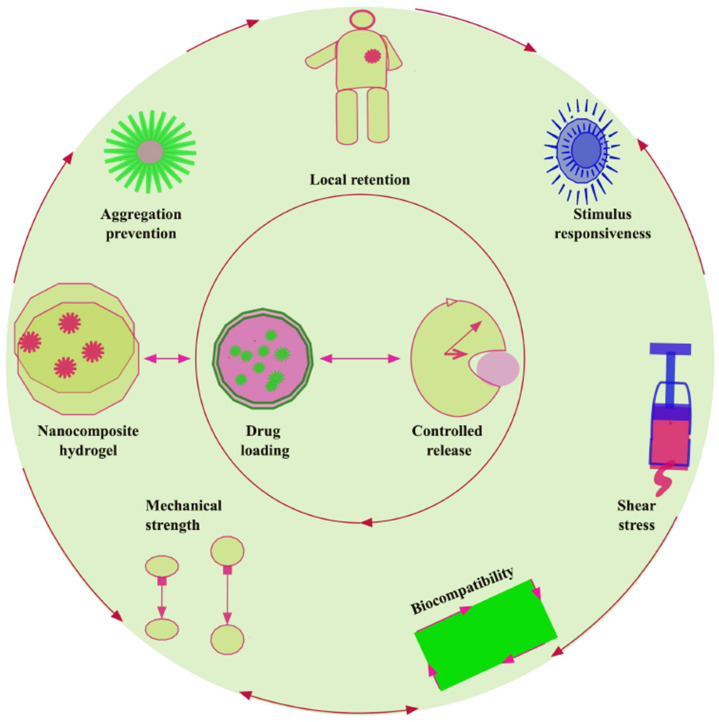
Dynamic hydrogel properties. Mechanical and functional properties of dynamic hydrogels with/without nanoparticles determine the cell aggregation inhibition, local retention of cells and drugs, controlled release of drugs depending on the mechanical strength, biocompatibility, shear stress, and stimulus-responsiveness nature of the hydrogels.

Hydrogels can prevent aggregation through several mechanisms. They can physically confine molecules within their porous structure, preventing them from clustering together. Additionally, the hydrophilic nature of hydrogels can hinder the aggregation of hydrophobic molecules by providing a water-rich environment. Furthermore, hydrogels can be designed with specific functional groups that interact with molecules to prevent aggregation through electrostatic or other non-covalent interactions [115,116] (Figure 4).

### 4.3. Control Release

Reloadable drug delivery depots offer therapeutic advantages when depot replacement is impractical, relying on highly specific drug–polymer interactions to enable repeated in vivo loading, selective drug targeting to distinct hydrogels, and tumor growth suppression. DNA base-pairing and bio-orthogonal click chemistries have demonstrated such reloading capabilities. Although mechanisms of drug release from hydrogels are well studied, in vivo release modeling remains limited due to unquantified parameters; integrating drug release kinetics with tissue transport models could enhance system design. Emerging bioelectronics and gene editing applications (e.g., CRISPR-Cas9, zinc-finger nucleases) highlight opportunities for hydrogel-based delivery, with potential for remote-controlled, responsive therapeutic release via integrated microelectronics, stretchable conductors, and reservoirs. With growing material diversity and application breadth, hydrogel drug delivery systems are poised to improve therapeutic scale, efficacy, and cost, with a lasting impact on healthcare [117] (Figure 4).

### 4.4. Shear-Thinning Hydrogels

Shear-thinning hydrogels are materials that behave like a liquid (low viscosity) under stress (like when being injected) and then return to a solid-like state (high viscosity) when the stress is removed. This reversible change in viscosity, called shear thinning, makes them useful for applications like injectable drug delivery and tissue engineering. They can flow easily through a syringe needle during injection but then solidify, providing support or a barrier at the target site. Shear-thinning hydrogels, which decrease in viscosity under shear and rapidly self-heal upon load removal, enable syringe preloading, injectable delivery, and improved retention compared to in situ crosslinking systems, reducing embolization risk.

Often formed via physical crosslinks, they may lack the mechanical stability of covalent gels; secondary crosslinking can enhance stability post-injection. These hydrogels have applications in drug delivery, tissue regeneration, mechanical bulking, and extrusion bioprinting, with crosslinking mechanisms including peptide or recombinant protein assembly, ionic or thermal interactions, electrostatics, supramolecular chemistry, and dynamic covalent bonds. Their injectability supports minimally invasive delivery and bioprinting, where parameters must preserve cell viability and structure resolution. As a model, guest–host supramolecular hydrogels were formed from β-cyclodextrin-modified and adamantane-modified hyaluronic acid, providing a platform for rheological characterization, injection force measurement, and cardiac tissue delivery [118] (Figure 4).

### 4.5. Mechanical Strength

Extracellular matrix (ECM) stiffness is a key regulator of cellular behavior, influencing migration, proliferation, and differentiation. In cancer, increased ECM stiffness is linked to tumor progression, metastasis, and the maintenance of cancer stem cell (CSC) populations, whereas softer matrices favor differentiation. Stiffer microenvironments promote CSC properties by modulating gene expression, cell division, and tumorigenesis [119]. Hydrogels for breast tumor models require a specific range of mechanical strength, often mimicking the stiffness of both healthy and cancerous breast tissue. They can range from ~2 kPa for normal tissue to upwards of 10 kPa for malignant tissue, with some tumor models reaching ~5.9 kPa. The mechanical properties of the hydrogel, such as stiffness and degradability, play a crucial role in how cancer cells behave, influencing their proliferation, migration, and response to therapies. A synthetic hydrogel scaffold composed of polyvinyl alcohol, collagen, and PLGA/polycaprolactone/gelatin nanofibers (PVA/COL/PPG) has been developed for breast reconstruction. The incorporated PPG nanofibers formed amide bonds with PVA and COL, enhanced by EDC/NHS crosslinking, which improved mechanical strength, structural integrity, thermal stability, and supported cell adhesion, proliferation, and 3D growth. These systems may also avoid excessive immune responses leading to implant rejection. However, long-term degradation behavior and translational validation of synthetic hydrogel prostheses in animal models remain to be comprehensively assessed [1] (Figure 4).

Composites combining alginate with gelatin and cellulose nanocrystals (CNCs) enhance mechanical strength, cell viability, and slow degradation. PEG-based dynamic hydrogels with surface-bound RGD maintain cell attachment, highlighting the importance of ligand presentation and molecular mobility [120]. Excessive molecular dynamics can disrupt mechanotransduction and impair adhesion; thus, a balance between dynamics and stability is necessary. Nanoreinforcements like CNCs improve hydrogel mechanical properties and support regenerative applications [121,122].

Cells remodel ECM and respond to mechanical cues, influencing gene expression via pathways such as YAP/TAZ. ECM stiffness modulates chromatin structure, with stiffer matrices promoting malignancy through epigenetic regulation. Dynamic hydrogels serve as platforms for mechanobiology and stem cell studies, with advanced sequencing techniques (e.g., ATAC-seq) revealing chromatin accessibility changes [123,124].

### 4.6. Stimuli-Responsive and pH-Sensitive Hydrogels

**Figure 5:**
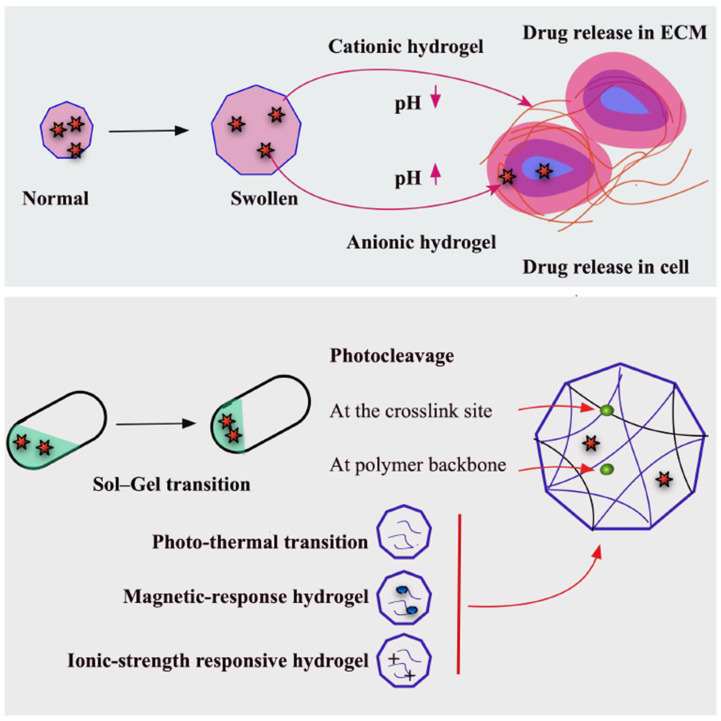
Stimulus responsiveness of dynamic hydrogels. Dynamic hydrogels are responsive to the pH, temperature, light, and ionic strength based on their chemical properties. Magnetic-responsive hydrogels can be created. pH-sensitive hydrogels usually swollen in the presence of cationic or anionic interactions, depending on the anions or cations present in the hydrogel molecules. Usually, drug releases in the ECM from cationic hydrogels, whereas in the cell from anionic hydrogels. Sol–Gel transition happens in photo, ionic, and magnetic response hydrogels. Hydrogel molecules will cleave at the crosslink site or at the backbone. Based on the hydrogel, biomaterials and their chemical structures determine the crosslink activities.

Cancer cells typically exhibit extracellular acidification and intracellular alkalization, with elevated intracellular pH enhancing glycolysis, promoting hypoxic adaptation, and driving proliferation. Exploiting this differential pH, pH-sensitive hydrogels have been engineered to achieve site-specific release of cytotoxic drugs within tumor microenvironments, thereby minimizing off-target toxicity. Incorporation of ionizable moieties such as amines, carboxylic acids, or imines impart pH responsiveness, enabling proton donation or acceptance depending on the environmental pH relative to the group’s pKa. Cationic hydrogels swell under acidic conditions (pH < pKa) due to protonation of amino/imine groups and electrostatic repulsion, while anionic hydrogels swell under alkaline conditions (pH > pKa) through the ionization of acidic groups. pH-induced conformational and solubility changes—via swelling/collapse, dissociation, or modulation of drug–matrix partition coefficients—govern release kinetics, which can be tuned by controlling the extent and ratio of structural transitions. Cationic hydrogels preferentially release drugs in acidic extracellular tumor environments, whereas anionic hydrogels are suited for controlled intracellular delivery [125] (Figure 5).

pH-sensitive hydrogels can also be prepared through polyelectrolyte complexation between anionic (e.g., alginate, dextran) and cationic (e.g., chitosan) polymers, avoiding potentially toxic chemical crosslinkers, or via the incorporation of acid-labile linkages that degrade selectively under acidic conditions. Chitosan, a natural, biodegradable polymer, is widely used but limited by poor solubility; derivatives such as N-trimethyl, N-carboxymethyl, and N-carboxyethyl chitosan (CEC) overcome this limitation. Similarly, CS/PVA hydrogels loaded with 5-fluorouracil retain the drug at physiological pH and release it at pH 5, minimizing healthy cell exposure.

A wide range of natural (e.g., alginate, cellulose, carrageenan, xanthan) and synthetic polymers (e.g., polyamines, acrylic derivatives, sulfonic acid derivatives, sulfonamides, pyridine and imidazole derivatives, PEG, PVP, PLA) have been used to develop pH-responsive systems. Beyond injectable applications, gastrointestinal pH gradients have been exploited for oral anticancer delivery. In vivo, this system improved survival, reduced hematopoietic apoptosis, and mitigated weight loss following γ-radiation exposure. These examples highlight the versatility of pH-sensitive hydrogels as smart platforms for targeted drug delivery, cancer therapy, and supportive treatment strategies [125] (Figure 5).

### 4.7. Photosensitive Hydrogels

Photosensitive hydrogels undergo chemical or physical alterations upon exposure to light (UV, visible, or near-infrared [NIR]), enabling spatially and temporally controlled drug release. Light triggers processes such as free-radical polymerization, photocleavage, isomerization, or volume changes via swelling/shrinkage. Three main strategies are used for light-responsive hydrogel design: (i) photochemical cleavage of crosslinks or polymer backbones, (ii) photo-induced isomerization altering crosslink density, charge, or hydrophilicity, and (iii) incorporation of photothermal agents inducing phase transitions in thermo-responsive hydrogels. Photoisomerization typically yields reversible systems, while photocleavage produces irreversible changes. Common photocleavable moieties include ruthenium, coumarin nitrophenyl, and o-nitrobenzyl derivatives, whereas azobenzene and spiropyran enable isomerization. Photothermal agents include porphyrins, cyanines, metallic nanoparticles (gold, silver, oxides), and carbon-based nanomaterials [126,127] (Figure 5).

NIR-responsive hydrogels are particularly attractive for biomedical use due to deep tissue penetration and low intrinsic toxicity, though overheating remains a concern. For example, NIR irradiation induces gel-to-sol in black phosphorus nanosheets in agarose hydrogels through transition via localized heating, enabling controlled doxorubicin release and tumor eradication in vivo, with both components being FDA-approved or metabolically safe. NIR-induced heating (>7 °C) exerts photothermal activity in a hyaluronic acid–dopamine hydrogel crosslinked with sodium selenite and loaded with indocyanine green (ICG) and, combined with Se’s pro-oxidant effect, significantly inhibits breast tumor growth in vitro and in vivo without toxicity [126,127].

Photodynamic therapy (PDT) hydrogels incorporate photosensitizers to generate reactive oxygen species upon irradiation. For example, Zn-phthalocyanine (ZnPc) in PEGDA-based hydrogels acts as both a photo-initiator and photosensitizer, generating singlet oxygen (^1O_2_) under NIR light and reducing HeLa cell viability, with potential for combinational chemotherapy. TiO_2_ serves as a photo-initiator and photosensitizer, with methylene blue enhancing ROS production in PEGDA hydrogels with methylene blue-sensitized TiO_2_ nanocrystals [126,127].

Dual-mode photothermal–photodynamic hydrogels have also been developed, such as an agarose-based system containing sodium humate (photothermal agent), chlorin e6 (photosensitizer), and MnO_2_ (H_2_O_2_ decomposition and hypoxia modulation). NIR irradiation increases temperature, degrades the agarose to release H_2_O_2_, catalyzes O_2_ generation, and produces ^1O_2_, achieving 93.8% tumor growth inhibition in vivo without systemic toxicity. These multifunctional hydrogels hold strong potential for tumor microenvironment modulation and synergistic cancer therapy [126,127] (Figure 5).

### 4.8. Magnetic and Ionic Strength Hydrogels

Beyond temperature, pH, and light, other stimuli such as magnetic fields and ionic strength can also regulate the properties of stimuli-responsive hydrogels. Magnetic-sensitive hydrogels typically incorporate iron oxide nanoparticles, which generate localized heat under an external magnetic field, enabling thermal ablation and, when combined with thermo-sensitive hydrogels, synergistic chemo-thermal therapy. For example, chitosan–PEG hydrogels with ferromagnetic vortex-domain iron oxide nanorings (FVIOs) and doxorubicin achieved near-complete tumor growth inhibition 21 days post-surgery via combined chemotherapeutic and thermal effects. Similarly, PEGylated Fe_3_O_4_/α-cyclodextrin hydrogels co-loaded with paclitaxel and doxorubicin exhibited magnetocaloric gel-to-sol transition for localized release, improving survival and reducing tumor recurrence in vivo [125,128,129].

Ionic strength-sensitive hydrogels, produced from ionizable or zwitterionic polymers (e.g., alginate, gellan gum, carboxymethyl dextran, polyacrylic acid, sulfobetaines, polypeptides), undergo conformational changes in response to ions such as K^+^, Na^+^, and Ca^2+^. In poly(L-glutamic acid-co-L-lysine) hydrogels loaded with doxorubicin, increased ionic strength shields electrostatic interactions between NH_3_^+^ and COO^−^ groups, promoting swelling and drug release. These systems often exhibit combined pH and enzymatic responsiveness, enhancing their multifunctional potential in cancer therapy [125,128,129] (Figure 5).

### 4.9. Dual-Responsive Hydrogels

Dual-responsive hydrogels, particularly thermo- and pH-sensitive systems, are gaining prominence for biomedical applications due to their multifunctionality and site-specific drug delivery potential (Figure 5). In cancer therapy, such systems can be injected in sol form, undergo in situ gelation at body temperature, and release drugs preferentially at acidic tumor pH. For example, chitosan/poly (N-isopropylacrylamide-co-itaconic acid) hydrogels loaded with doxorubicin exhibited faster release at pH 5.5 than pH 7.4 due to chitosan protonation, enhancing cytotoxicity against MCF-7 breast cancer cells. Similarly, lysine-modified poly (vinylcaprolactam) nano-hydrogels conjugated with doxorubicin via Schiff base linkage achieved maximal release at 40 °C and pH 5 [125,130].

A crosslinker-free poly (ethylene glycol) methyl ether methacrylate/acrylic acid hydrogel demonstrated pH- and temperature-dependent swelling for oral 5-fluorouracil delivery in colorectal cancer, retaining the drug at gastric pH and releasing it at intestinal pH (>6), thereby avoiding premature release and maintaining activity against HepG2 cells. More complex designs include co-delivery systems of granzyme B and docetaxel in pH-sensitive micelles (PGA-PLH) embedded in thermo-sensitive hydrogels (mPEG-b-PELG), enabling enzyme-triggered micelle release, tumor penetration, pH-mediated drug unloading at pH 5.5, and synergistic antitumor efficacy in vivo.

Beyond pH–thermo combinations, other dual-responsive systems have been reported. A photo- and thermo-responsive multicompartment hydrogel (PNBOC-b-PNAM-b-PNIPAM) loaded with gemcitabine and doxorubicin undergoes sol-to-gel transition upon administration, with UV-triggered drug release via micelle core crosslinking and hydrophobic-to-hydrophilic transition, enabling spatiotemporally controlled combination therapy [125,130].

Enzyme-responsive hydrogels degrade or release cargo in response to disease-associated enzymes (e.g., matrix metalloproteinases). DNA/RNA-responsive hydrogels employ nucleic acid hybridization for targeted assembly/disassembly, enabling precise drug release or biosensing. Aptamer-functionalized hydrogels selectively bind molecules, producing detectable structural or colorimetric changes useful for diagnostics [131,132]. These smart hydrogels integrate sensing, targeting, and therapeutic functions, providing powerful platforms for personalized medicine, biosensing, and responsive therapeutic delivery.

## 5. Hydrogel Applications in Breast Tissue Regeneration

### 5.1. Scaffold Provision

Hydrogels serve as scaffolds to be considered highly in breast tissue engineering. Natural hydrogels offer superior biomimicry and support for cell viability, effectively creating 3D-printed architectures replicating the native tissue ECM [133] (Table 2) (Figure 2).

When combined with bioactive molecules promoting adipogenesis and angiogenesis, natural hydrogels facilitate tissue integration and regeneration [134]. Natural materials such as decellularized extracellular matrix (dECM), gelatin, and sodium alginate (SA) offer excellent biocompatibility and minimal immune rejection due to their origin from biological sources [51].

In contrast, synthetic hydrogels afford high customizability and mechanical durability through advanced fabrication methods, allowing tailoring of porosity, degradation rates, and surface characteristics to meet clinical demands for stability and load bearing. To overcome the limitations of each material type, composite scaffolds combining natural and synthetic components are often developed to achieve an optimal balance of mechanical integrity and biological functionality [135]. Despite advances, natural hydrogels remain limited by scalability, mechanical robustness, and immune reactions, while synthetic hydrogels require improvements in biocompatibility and biodegradability (Table 2).

### 5.2. Surgical Reconstruction Strategies

Breast function and aesthetics restoration post-lumpectomy or mastectomy involve diverse surgical options tailored to patient needs. Tissue flaps and fat grafting are limited, especially post-lumpectomy. Mastectomy reconstruction choices include synthetic implants and autologous tissue flaps, each with pros and cons—implants offer simpler surgery but risks such as capsular contracture, while autologous flaps provide natural outcomes at the expense of longer recovery and donor site morbidity. Common techniques include TRAM, latissimus dorsi, DIEP, and newer methods like PAP and LTP flaps. Emerging hydrogel-based approaches to support fat transplantation and tissue regeneration show promise but have yet to reach clinical standard.

Human breast tissue mainly comprises vascularized adipose tissue, with adipocytes occupying the most volume. Successful breast restoration requires scaffolds supporting adipocyte survival and proliferation. Natural ECM-derived scaffolds such as collagen and hyaluronic acid, including decellularized tissues, mimic native microenvironments, fostering integration. These matrices serve as “soil” for regenerative “seeds” including preadipocytes, smooth muscle cells, and stem cells. Notable methods include collagen microfiber bio-inks encapsulating mature adipocytes, adipose-derived stem cells (ADSCs), and endothelial cells (HUVECs) to produce vascularized constructs, and bottom-up techniques assembling microtissues on collagen microgels to enhance adipose regeneration and neovascularization. These approaches offer scalable strategies for functional breast tissue reconstruction [136,137].

## 6. Hydrogel Application in Breast Tumor Models

### 6.1. Three-Dimensional Tumor Model

Three-dimensional hydrogel systems mimic the breast tumor microenvironment (TME) by incorporating bioactive ECM components or analogs like chitosan, alginate, hyaluronic acid, cellulose, collagen, gelatin, and silk fibroin. These platforms facilitate studies of cell–cell and cell–matrix interactions, gene expression, and tumor heterogeneity. Collagen type I, abundant in breast tissue, is crucial for ECM structure and acini maintenance. Bioprinting with collagen bio-inks improves tissue fidelity and biological relevance, enabling vascularized tumor organoids useful for drug screening. Functionalized hydrogels, including recombinant spider silk with RGD motifs or elastin-like recombinamer (ELR)-based gels with MMP-degradable sequences, enhance cell adhesion and drug resistance, though some non-human ECM sources may limit clinical translation [45,51,138] (Figure 2).

Co-culturing cancer cells with stromal components (fibroblasts, macrophages) in hydrogel scaffolds models tumor heterogeneity and chemoresistance. Alginate cryogels incorporating macrophages create immune-like microenvironments. Microfluidic bioprinted platforms allow investigation of tumor–normal cell interactions and migration. Patient-derived cells or tissues cultured in PEG–heparin or decellularized scaffolds retain phenotypes and drug resistance, advancing personalized cancer modeling for precision medicine and drug screening [15,30,139].

Three-dimensional hydrogel cultures better recapitulate the TME than two-dimensional cultures, improving drug testing relevance. Patient-derived organoids (PDOs) retain tumor genotype and phenotype and exhibit clinically relevant chemoresistance, enabling personalized therapy testing and biobanking. Studies confirm strong correlations between PDO drug responses and patient outcomes, supporting precision oncology applications [140]. Enhanced models incorporate immune components, enabling immunotherapy and microbiome interaction studies. PDOs also facilitate long-term hormone therapy research in estrogen receptor-positive breast cancer. Limitations include sample availability from biopsies, heterogeneity, and incomplete ECM mimicry, underscoring the need for improved hydrogel formulations and integration of TME components to enhance model fidelity [141,142].

### 6.2. Emerging Technologies: 3D Bioprinting and Self-Folding Hydrogels

Three-dimensional bioprinting techniques—extrusion, inkjet, stereolithography, microfluidics—enable precise fabrication of complex hydrogel architectures mimicking native tissues. Dynamic hydrogels’ injectability and shear thinning facilitate high-resolution printing with stimulus-responsive crosslinking for spatiotemporal control. Challenges include mechanical robustness, biocompatibility, and controlled degradation aligned with tissue healing [143]. Self-folding hydrogels form complex 3D structures, supporting cell adhesion, migration, and tissue development. Applications extend to microgripping for minimally invasive surgeries and shape-memory drug delivery systems enabling controlled, targeted release. Integration into surgical tools enhances flexibility and navigation [144]. Dynamic hydrogels allow spatiotemporal control of mechanical and biochemical cues, facilitating detailed studies of chemotaxis, haptotaxis, and mechanotransduction under physiologically relevant forces [145].

## 7. Hydrogels in Breast Cancer Therapy

### 7.1. Drug Delivery and Immunotherapy

Hydrogels offer controlled drug release capabilities accommodating chemotherapeutics, immunosuppressants, and immune stimulants. Available in macro-, micro-, and nanoforms, they support diverse administration routes, including intravenous, transdermal, oral, pulmonary, and localized implantation (Figure 3) [146]. Stimuli-responsive hydrogels react to pH, temperature, or redox conditions for site-specific release, enhancing therapeutic efficacy and sparing healthy tissue. Injectable hydrogels facilitate localized delivery to tumor resection sites with minimal mechanical disruption [147]. Incorporation of silicate nanoparticles broadens functional applications. Chitosan-based hydrogels have been widely studied for localized delivery and mucoadhesiveness. Examples include methacrylated glycol chitosan hydrogels extending DNA/doxorubicin release, reducing lung metastasis and improving survival; 3D sponges loaded with cisplatin–chitosan–calcium alginate microparticles showing antitumor and regenerative effects [98,148]. Silica nanoparticles embedded in Pluronic F-127/hyaluronic acid hydrogels show synergistic cytotoxicity with nitric oxide donors. Silk fibroin hydrogels support doxorubicin delivery, adipose stem cell differentiation, and vascularization, targeting triple-negative breast cancer and restoration. Cellulose nanofiber hydrogels loaded with 5-fluorouracil demonstrate anticancer effects and stem cell support. Succinic anhydride-based hydrogels co-delivering paclitaxel and mifepristone inhibit metastasis biomarkers effectively [149,150].

Dynamic hydrogels derived from natural or synthetic biomaterials have evolved with nanocomposite advances. Embedding nanoparticles enhances mechanical strength and controlled drug delivery, facilitating multidrug therapies for antibiotic-resistant infections, cancer, and cardiovascular diseases. Applications include dual delivery systems promoting osteogenic differentiation and bone regeneration. Future work targets improved self-healing, adhesiveness, and durability [72,151].

Drug release profiles depend on drug hydrophobicity, nanocarrier properties, and hydrogel degradability (Figure 3) [152]. Chemically crosslinked nanocomposites formed via thiol–Michael or amine–epoxy additions enable sequential drug release and combinatorial therapies [153]. Examples include Pluronic nanoparticles releasing BIO rapidly and PLGA microspheres sustaining VEGF delivery for stroke recovery [154,155]. Host–guest gelatin hydrogels and MgFe nanohybrids achieve slow ion/drug release, promoting osteogenesis and bone repair. Dispersion of nanocarriers is optimized via amphiphilic stabilizers and electrospinning. Stimuli-triggered drug release mechanisms respond to pH, ionic strength, temperature, or light, enabling precise on-demand delivery [156,157]. Acid-responsive hydrogels incorporate cleavable bonds (imine, catechol–catechol) for enhanced mechanical properties and pH-sensitive drug release (Figure 5). Redox-responsive hydrogels leverage glutathione differences in tumor environments to trigger thiol-exchange reactions for targeted delivery [158,159]. Photosensitive and photothermal nanomaterials (gold nanoparticles, carbon nanomaterials, metal–organic frameworks) integrated into hydrogels enable non-invasive photothermal and photodynamic therapies producing heat or reactive oxygen species for antibacterial and anticancer effects (Figure 5) [160,161].

Multi-stimuli-responsive nanocomposites exhibit enhanced therapeutic precision, with systems incorporating ROS- and light-responsive nanoparticles for synergistic tumor inhibition and metastasis prevention [162,163]. Temperature-responsive nanocomposite hydrogels employ phase transitions to regulate drug release, combining materials like PNIPAM, gelatin, and PLGA-PEG-PLGA. Triple-responsive hydrogels integrate pH, redox, and temperature triggers for combinational cancer therapy [164,165].

Cancer immunotherapy strategies include monoclonal antibodies, checkpoint inhibitors, CAR T-cell therapy, cancer vaccines, and cytokine therapies. While clinical success in breast cancer is limited, hydrogels are gaining attention as immunotherapeutic delivery vehicles. Methacrylated glycol chitosan hydrogels can induce immunogenic cell death, augmenting immune responses alongside drug delivery. Chitosan nanoparticle hydrogels loaded with cisplatin and rituximab showed limited efficacy due to targeting challenges. Chitosan-based hydrogels delivering IL-12 eradicated triple-negative breast tumors and induced immune memory, indicating neoadjuvant potential [32,166]. Combination chemoimmunotherapy with pembrolizumab and cytotoxics improves survival in PD-L1+ patients but increases toxicity. Antibody–drug conjugates like trastuzumab–deruxtecan have shown remarkable clinical benefits in HER2+ tumors. Chitosan hydrogels allow controlled monoclonal antibody release, supporting integrated immunotherapy platforms [167,168].

### 7.2. Advanced Therapies and Photothermal Approaches

Hormone therapy remains clinically important. Temperature-sensitive hydrogels (e.g., Tam-Gel) enable controlled tamoxifen release responsive to body temperature, enhancing therapeutic precision [169]. Photothermal therapy (PTT), utilizing near-infrared light to generate localized heat, has been incorporated into hydrogels [170]. Injectable IR820/Mgel hydrogels achieve tumor ablation at >50 °C under NIR irradiation, preventing recurrence. Polysaccharide hydrogels loaded with BSA-MoS_2_ nanoflakes provide synergistic photothermal and photodynamic therapy [171]. Copper-induced hydrogels combining PD-L1 antibodies and nitric oxide donors enhance immune cell infiltration post-NIR exposure. Three-dimensional-printed polydopamine-modified scaffolds mimic breast tissue mechanics, supporting epithelial growth alongside PTT. Graphene-enhanced self-healing hydrogels combine chemotherapy and PTT with potent antitumor effects [172,173]. Curcumin-derived hydrogels protect the drug from degradation while selectively killing cancer cells, demonstrating dual therapy and reconstruction utility [174].

Despite innovations, hydrogel drug delivery systems are mostly preclinical. Clinical translation faces challenges including systemic application, long-term effects, degradation safety, and drug compatibility. Regulatory progress includes injectable hydrogels approved for prostate and urothelial cancers, with breast cancer-specific trials ongoing, such as RadiaAce for radiation dermatitis. Hydrogels with special extracellular proteins, for example, asporin might be incorporated to align the collagen fibers, inhibiting the cancer migration [175]. Analogous systems tested in other cancers hint at future approval pathways.

## 8. Translational Barrier from Bench to Bedside

Hydrogels have emerged as highly versatile biomaterials with broad potential for applications ranging from drug delivery and wound healing to tissue regeneration and cancer therapy. Their high-water content, tunable mechanics, and biocompatibility make them attractive as biomimetic extracellular matrix (ECM) analogs. Despite this promise, however, the clinical translation of hydrogels has been slow. Only a handful of hydrogel-based systems—such as certain ophthalmic gels, dermal fillers, and wound dressings—are commercially available, while many advanced formulations remain confined to laboratory settings. Several interrelated scientific, technical, regulatory, and economic barriers hinder the smooth translation of hydrogel technologies from bench to bedside [176].

One of the foremost challenges is the complexity of hydrogel formulations. Laboratory-developed hydrogels often rely on sophisticated chemistries, such as dynamic covalent bonds, supramolecular interactions, or multifunctional polymer blends. While these allow fine control over stiffness, degradation, and bioactivity in vitro, they present challenges in scalability and reproducibility. Batch-to-batch variations in polymer synthesis, crosslinking density, or biofunctionalization can significantly alter hydrogel performance. For instance, naturally derived hydrogels such as collagen, hyaluronic acid, or Matrigel suffer from source-dependent variability, limiting their consistency in clinical applications. Establishing robust manufacturing pipelines with Good Manufacturing Practice (GMP) compliance is difficult for complex hydrogel systems, making regulatory approval more challenging [177].

Although hydrogels are generally considered biocompatible, immunogenicity and host responses remain significant hurdles [178]. Residual crosslinkers, degradation byproducts, or non-natural chemical modifications can provoke inflammation, fibrosis, or immune rejection. Furthermore, hydrogels designed for in situ gelation (e.g., injectable systems using UV or chemical crosslinking) raise concerns about local cytotoxicity from photo-initiators or reactive species [179]. Even hydrogels with good short-term biocompatibility may induce chronic inflammatory responses over time, especially in long-term implants. Predicting these responses in humans remains difficult since small-animal models often fail to fully recapitulate human immune systems.

Hydrogels developed in vitro are typically optimized under static and controlled conditions. However, the in vivo environment is dynamic, enzymatically active, and mechanically demanding. Hydrogels often suffer from premature degradation, loss of integrity, or uncontrolled swelling, which compromise therapeutic function. For example, hydrogels used in cartilage regeneration must withstand repetitive mechanical stress, while injectable tumor models must remain structurally intact long enough to support drug release or cell culture [179,180].

A critical translation barrier lies in hydrogel–host integration. For tissue engineering, hydrogels must support cell infiltration, vascularization, and remodeling, while avoiding fibrous encapsulation. For drug delivery, they must release payloads in a predictable manner without burst effects or premature leakage. However, the complex interplay between hydrogel degradation, local tissue remodeling, and cellular responses often produces unpredictable outcomes. In breast cancer models, for instance, hydrogels that mimic extracellular stiffness can influence drug resistance; translating this into a therapeutic platform requires careful control of how the material interacts with both tumor and stromal cells.

Hydrogels often fall into a gray zone of regulatory classification, being both medical devices and drug carriers. This duality complicates approval pathways, as products may be regulated under device, biologic, or combination product frameworks depending on their function. Additionally, regulatory bodies demand extensive toxicology, pharmacokinetics, and long-term safety data. For advanced hydrogels with responsive or dynamic properties, regulators face uncertainty about how to evaluate stability and failure risks. This lack of clear guidelines can deter industrial investment and delay clinical progression [181].

Many hydrogels are easy to fabricate in the lab but difficult to manufacture at clinical scale. Challenges include the following: (a) Sterilization: Conventional methods such as gamma irradiation, autoclaving, or ethylene oxide can damage sensitive hydrogel networks or bioactive components. (b) Shelf life and storage: Hydrogels are often unstable in hydrated form, requiring cold-chain storage or lyophilization strategies that may alter structure, and (c) Scalability: Processes such as microfluidic encapsulation, 3D bioprinting, or multi-step functionalization are difficult to scale economically [182].

Thus, moving from milligram-scale prototypes to kilogram-scale GMP production is non-trivial.

Finally, the translation of hydrogels is limited by cost, market competition, and reimbursement pathways. Many synthetic hydrogels involve expensive polymers, photo-initiators, or functional peptides, raising production costs beyond what is feasible for routine clinical use. Furthermore, hospitals and clinicians may prefer simpler, already-approved materials (e.g., sutures, synthetic meshes) unless hydrogel systems provide clear, measurable advantages in patient outcomes. Economic barriers are compounded by the need for long, costly clinical trials.

## 9. Discussion

Tumor progression involves dynamic interactions between cancer cells and the tumor microenvironment (TME), including stromal fibroblasts, immune infiltrates, extracellular matrix (ECM) components, vascular networks, and soluble signaling molecules. Understanding these multifaceted interactions requires experimental models capable of accurately recapitulating both cellular and microenvironmental complexity.

Dynamic hydrogels have emerged as powerful platforms to investigate the complex and evolving tumor microenvironment (TME) in breast cancer. Unlike static hydrogels, which provide a constant mechanical and biochemical milieu, dynamic hydrogels can mimic the time-dependent remodeling, stiffening, and viscoelastic behavior that are central to tumor progression [183] (Figure 2, Table 2). Breast tumors are characterized by progressive extracellular matrix (ECM) stiffening, increased deposition of collagen and hyaluronic acid, and elevated activity of matrix metalloproteinases (MMPs). These features are critical determinants of tumor cell proliferation, invasion, metastasis, and therapeutic resistance (Figure 1). Dynamic hydrogels are uniquely suited to replicating these aspects, offering a more physiologically relevant and predictive platform compared to traditional static systems [184] (Table 2).

Dynamic hydrogels have demonstrated significant advances in three major areas of breast tumor research. Firstly, they enable detailed mechanobiology studies, allowing researchers to decouple stiffness from viscoelasticity and to examine how stress relaxation influences epithelial–mesenchymal transition (EMT), stemness, and metastatic potential. Secondly, they provide a controllable environment for co-culture studies with cancer-associated fibroblasts (CAFs), immune cells, and endothelial cells, thereby illuminating how stromal crosstalk drives desmoplasia and immune exclusion. Thirdly, dynamic hydrogels support therapeutic applications, such as modeling-acquired drug resistance, testing combination therapies under evolving matrix conditions, and even serving as injectable therapeutic depots or scaffolds for local drug delivery [185,186] (Table 2, Figure 2).

Nonetheless, hydrogel-based research has already influenced therapeutic design. Stiff substrates better predict chemoresistance than conventional two-dimensional cultures, reinforcing the importance of physiologically relevant models. Hybrid hydrogels combining natural and synthetic polymers show promise in balancing bioactivity and mechanical tunability, while dynamic and magnetic hydrogels add external control and flexibility [183] (Table 1 and Table 2 and Figure 2). Their potential extends beyond tumor modeling to regenerative medicine, where multifunctional hydrogels could simultaneously support tissue reconstruction and deliver localized therapy. Future progress will depend on developing spatiotemporally dynamic systems, integrating hydrogels with microfluidics for greater physiological realism, addressing immunological concerns, and standardizing fabrication for reproducibility. Nevertheless, despite their promise, several limitations hinder widespread adoption. Dynamic hydrogels often involve complex chemistries that are difficult to standardize, raising concerns about reproducibility and scalability. Moreover, the balance between mechanical adaptability and structural integrity remains a challenge: hydrogels that remodel easily in vitro may degrade too rapidly in vivo [187]. From a translational perspective, many dynamic systems incorporate photo-initiators, reactive linkers, or supramolecular components with uncertain safety profiles, complicating clinical translation. The high degree of tunability, while scientifically valuable, increases experimental variability, demanding careful experimental design and thorough rheological characterization [23].

Taken together, dynamic hydrogels represent a transformative step in breast tumor modeling, bridging the gap between simplified static systems and the complexity of in vivo biology. Their ability to capture the temporal and spatial evolution of the breast TME positions them as indispensable tools for mechanistic studies and preclinical drug development, though critical barriers must be addressed before they can fully realize their translational potential [188].

Looking ahead, the future development of dynamic hydrogels for breast tumor models will likely focus on interrelated directions. Promising trajectory is the design of dual-network hydrogels that integrate both permanent and reversible crosslinks. Such hybrid systems could provide a stable baseline mechanical framework while retaining tunable dynamic features, balancing robustness with adaptability. In breast cancer modeling, these systems could capture early tumor initiation on a soft baseline while allowing controlled stiffening to simulate desmoplastic progression [12]. Hybrid systems also provide improved reproducibility compared to purely supramolecular or reversible chemistries [34] (Figure 2).

Dynamic hydrogels are poised to play a central role in personalized oncology. By integrating patient-derived breast tumor cells, CAFs, and immune infiltrates into adaptable hydrogel matrices, researchers can recreate individualized tumor niches that better predict therapeutic response. For example, tuning hydrogel stiffness and ligand composition to match the ECM profile of a patient’s tumor biopsy could enable ex vivo drug screening platforms [97]. Such biopsy-on-a-chip models, supported by dynamic hydrogels, may accelerate personalized treatment selection in triple-negative breast cancer (TNBC), where therapeutic options are limited [14]. The convergence of 4D bioprinting and dynamic hydrogel chemistry represents another frontier. Photopatternable and light-responsive hydrogels can create spatiotemporal gradients of stiffness, ligand density, and growth factors [84] (Figure 4 and Figure 5). For breast tumor models, this capability is essential to reproduce invasive fronts, hypoxic niches, or perivascular regions within the same construct. Combining bioprinting with dynamic hydrogels will enable fabrication of multicellular, compartmentalized tumor tissues that evolve over time, bringing in vitro models closer to in vivo architecture. Dynamic hydrogels will increasingly be coupled with microfluidic devices to mimic perfusion, nutrient gradients, and interstitial flow. In breast cancer-on-chip systems, such integration could simulate vascular stiffening, immune infiltration, or drug penetration barriers in real time [14,40]. Importantly, dynamic hydrogels allow these microenvironments to evolve dynamically, which is critical for modeling processes such as metastasis to bone or lung. These integrated systems may provide more reliable preclinical testing platforms, reducing reliance on animal models and accelerating drug discovery. For dynamic hydrogels to move beyond research tools into clinical application, emphasis must shift toward translation-oriented design. This includes selecting polymers and crosslinking chemistries with established safety profiles, simplifying synthesis routes, and ensuring scalability under GMP conditions. Furthermore, early engagement with regulatory bodies will be essential to define safety, degradation, and quality control standards for hydrogels with dynamic or stimuli-responsive properties. Collaborative efforts among materials scientists, clinicians, and regulatory agencies are needed to pave clear approval pathways for therapeutic hydrogel applications.

## 10. Conclusions and Future Perspectives

Dynamic hydrogels hold the potential to revolutionize both tumor biology research and therapeutic innovation in breast cancer. By enabling precise, time-resolved control over the cellular microenvironment, they open opportunities to dissect mechanisms of tumor progression, metastasis, dormancy, and therapy resistance that static systems cannot capture. Enthusiasm must be balanced with critical recognition of the limitations. Hydrogels remain oversimplified representations of the dynamic, heterogeneous ECM, often failing to replicate the stiffness gradients and temporal evolution of tumors. Reproducibility and scalability in hydrogel synthesis are further challenges, with subtle variations significantly altering mechanical behavior. Translational hurdles, including immune responses and long-term stability, pose additional obstacles for clinical applications. Moreover, the assumption that stiffness directly drives malignancy is reductive; cellular responses vary across cancer types, and stiffness interacts with other microenvironmental factors such as hypoxia and immune infiltration.

In the near term, dynamic hydrogels will be most impactful as research platforms for mechanobiology, stromal interactions, and drug resistance studies. In the longer term, as manufacturing and regulatory hurdles are addressed, they may evolve into clinically deployable scaffolds for localized drug delivery, regenerative reconstruction after tumor resection, and even immune modulation in the tumor bed.

Dynamic hydrogels represent a critical leap forward in breast tumor modeling. They bridge the divide between the reductionist clarity of static materials and the physiological complexity of in vivo tissues. While significant barriers to translation remain, continued interdisciplinary innovation—combining advanced polymer chemistry, biofabrication technologies, and clinical insight—will be key to unlocking their full potential. The future of breast cancer research and therapy will increasingly depend on such dynamic, adaptive biomaterials that can evolve in step with the disease itself.

## Data Availability

Not applicable.
